# Timing of intubation, beds in intensive care and inter-hospital transfer: rings of a complex chain during pandemic conditions

**DOI:** 10.1186/s13054-022-03925-1

**Published:** 2022-02-12

**Authors:** Filippo Sanfilippo, Luigi La Via, Giuseppe Carpinteri, Marinella Astuto

**Affiliations:** 1 Department of Anaesthesia and Intensive Care, A.O.U. Policlinico-San Marco, Via Santa Sofia 78, 95100 Catania, Italy; 2 Department of Emergency Medicine, A.O.U. Policlinico-San Marco, Via Santa Sofia 78, 95100 Catania, Italy

Dear Editor,

Gonzalez et al. published a prospective study focusing on timing of intubation and outcome of patients with coronavirus disease (COVID-19) [1]. The authors considered early intubation if this occurred within the first 48 h from the first respiratory support, and compared this strategy (*n* = 140) with a group that received delayed intubation (*n* = 65). The authors reported that delayed intubation caused a significant increase in hospital mortality (hazard ratio 2.4 in multivariate analysis); moreover, survivors in the delayed intubation group had worse pulmonary sequelae as evaluated by CT scan and DLCO.

These results are interesting, and other recent evidence is pointing towards benefits of early intubation in COVID-19 patients [2], in contrast with previous findings published by the journal [3]. Indeed, a recent meta-analysis investigated this topic including 12 studies and almost 9000 COVID-19 patients. The pooled evidence showed a trend towards increased all-cause mortality in patients receiving early invasive mechanical ventilation (45.4%) as compared with those with late intubation (39.1%; Risk Ratio 1.07, 95% CI 0.99–1.15, *p* = 0.08) [3].

How to interpret such discrepancies? We believe that timing of intubation is only one of the rings of a complex chain during pandemic conditions (Fig. 1). Indeed, while emergency and critical care physicians strive to deliver the most appropriate treatment for COVID-19 (and non COVID-19!) patients, the decision to intubate early should be always balanced with other local factors, and in particular with the availability of intensive care unit (ICU) beds.

**Fig. 1:**
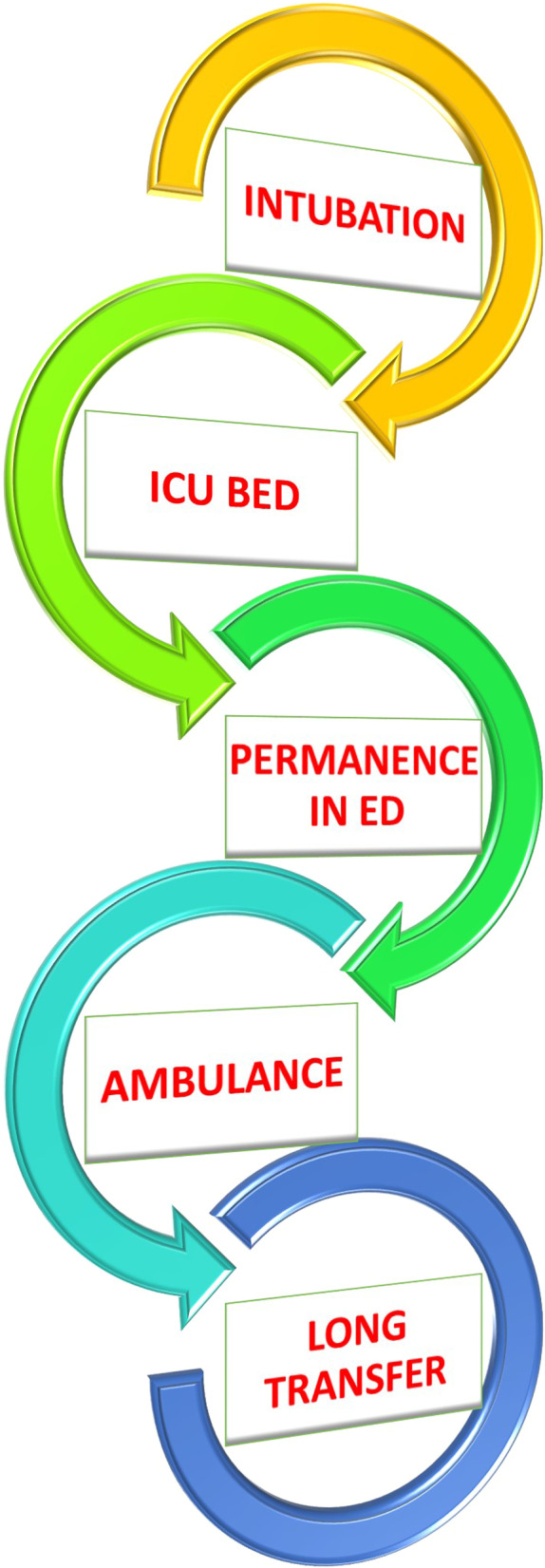
Rings of a complex chain in decision-making for the timing of intubation during pandemic conditions. *ED* emergency department, *ICU* intensive care unit

The surge in hospital admissions caused by the current pandemic wave is causing serious issues of bed availability in several hospitals. It is not uncommon that the closest ICU bed available is hundreds of kilometres away, in a different province/region. Thus, the decision to intubate may sometimes imply the need for a long inter-hospital transfer. To make things more complex, the prompt availability of specialized ambulances for transfer of critically ill patients is seriously challenged too. Indeed, ambulance services are overloaded by demands and rows of ambulances with COVID-19 patients waiting outside the emergency departments are reported with almost daily frequency in each country. Under certain emergency circumstances, even military healthcare teams have been deployed to assist the ambulance service [4]. Therefore, it frequently happens that intubated patients remain in the Emergency Department (ED) for several hours before the transfer can be accomplished. Finally, even if an ICU bed has been found and a specialized ambulance is available, the risks associated with long inter-hospital transfers of COVID-19 patients should not be underestimated [5].

While we applaud the authors for their well-conducted study, we think that all the analyses performed on timing of intubation of COVID-19 under challenging pandemic conditions should also account for the time occurring from intubation to the effective ICU admission. Only at this time, specialized critical care treatments may be fully delivered with a better staff-to-patient ratio, the use of high-performing ventilators and adequate monitoring systems. In summary, it is possible that patients exposed to long inter-hospital transfer and/or delayed ICU admission may not similarly benefit of a strategy of early intubation.

## Data Availability

Not applicable.

## Abbreviations

COVID-19: Coronavirus disease 2019
ED: Emergency Department
ICU: Intensive Care Unit
